# Living in disadvantaged neighborhoods linked to less intervention for severe aortic stenosis

**DOI:** 10.1038/s41598-024-52660-w

**Published:** 2024-02-28

**Authors:** Chirag Ram, Sameh Yousef, Wei-Guo Ma, Ishani Vallabhajosyula, Saket Singh, Ritu Agarwal, Rita K. Milewski, Roland Assi, Prakash A. Patel, Matthew Williams, Arnar Geirsson, Prashanth Vallabhajosyula

**Affiliations:** 1 Division of Cardiac Surgery, Yale Aortic Institute, Yale School of Medicine, 330 Cedar Street BB204, New Haven, CT 06520 USA; 2 Joint Data Analytics Team, Information Technology Service, Yale University, New Haven, USA; 3 Division of Cardiac Anesthesiology, Yale School of Medicine, New Haven, CT USA; 4 Vanderbilt University School of Medicine, Nashville, TN USA

**Keywords:** Cardiology, Risk factors

## Abstract

To investigate the association between area deprivation index (ADI) and aortic valve replacement (AVR) in patients with severe aortic stenosis (AS). Patients aged 40–95 years with severe AS confirmed by echocardiography were included. The 9-digit zip code of patient residence address was used to identify the ADI ranking, based on which patients were divided into 5 groups (with Group E being most deprived). The rates of AV intervention were compared among 5 groups using competing risks analysis, with death as a competing event. We included 1751 patients with severe AS from 2013 to 2018 followed for a median 2.8 (interquartile range, 1.5–4.8) years. The more distressed ADI groups tended to be younger (*P* = 0.002), female (*P* < 0.001), and of African American race (*P* < 0.001), have higher presentation of sepsis (*P* = 0.031), arrhythmia (*P* = 0.022), less likely to have previous diagnosis of AS (*P* < 0.001); and were less likely to undergo AVR (52.5% vs 46.9% vs 46.1% vs 48.9% vs 39.7%, *P* = 0.023). Using competing risk analysis, the highest ADI group (E) were the least and the lowest ADI group (A) the most likely to undergo AVR (Gray’s test, *P* = 0.025). The association between ADI ranking and AVR rates was influenced by sex and race. Within group analysis, there was significant association between race and AVR (Gray’s test, *P* < 0.001), and between sex and AVR (Gray’s test, *P* < 0.001). Patients with severe AS living in more deprived neighborhoods were less likely to undergo aortic valve interventions, which was influenced by female gender, and African American race.

## Introduction

Calcific aortic stenosis (AS) is the most common valvular heart disease in Western countries with severe stenosis affecting 3% of the population older than 75 years of age^1,2^. The incidence is increasing due to the aging of the population and the high burden of cardiovascular risk factors^1,3–5^. Without intervention, the disease is associated with severe outcomes including mortality, heart failure (HF), and poor quality of life. Clinical research led to improvements in disease management. The invention of transcatheter aortic valve replacement (TAVR) for patients with high surgical risk and the increasing intervention in asymptomatic patients with severe AS has helped improve outcomes^6^. Yet, the rate of timely intervention in patients with severe symptomatic AS has remained significantly low, 57% at the highest estimates^7^. Few sociodemographic factors, such as gender and racial disparities, have been studied in the context of disparity in the treatment of AS^8^, however, data on the effect of other sociodemographic determinants of health on the outcomes of aortic stenosis needs to be better understood in order to implement comprehensive, inclusive care for AS patients.

Recent data show neighborhood distress was associated with worse survival after combined coronary artery bypass graft (CABG) and aortic valve replacement (AVR) among Medicare beneficiaries^9^ and after TAVR^10^. As a result, area deprivation index (ADI) has been proposed as a metric that represents many of the social determinants of health and was validated in coronary heart disease (CAD) and peripheral arterial disease (PAD)^11–13^. ADI is calculated based on data from the American Community Survey (2011–2015) and considers 17 indicators from the US census on poverty, education, housing, and employment^14^. Every neighborhood represented by the 9-digit zip code is assigned a ranking score from 0 to 10 at the state level: the higher the ADI ranking, the more deprived is the neighborhood. The neighborhood disadvantage, as expressed by ADI, is gaining attention as an important determinant of cardiovascular outcomes, and access to timely quality medical care.

To better understand this correlation, we aimed to investigate the neighborhood distress represented by the area deprivation index (ADI) and its association with patterns of presentation, comorbidity profiles, interventions, and mortality among patients with severe AS in a single health care system. This may guide targeted interventions to mitigate the consequences AS in these vulnerable groups.

## Materials and methods

### Database and patient population

This is single center, retrospective review of patients who underwent echocardiography at Yale-New Haven Health System (YNHHS).

YNHHS is the largest healthcare system in the state of Connecticut and includes multiple hospitals, outpatient centers, and related health services throughout Connecticut as well as New York and Rhode Island^15^.

Patients with valvular heart disease captured within the system are eventually referred to a member the cardiac surgery team for surgical intervention or to the structural heart team for evaluation of possible trans-catheter intervention. Echocardiographic data (transthoracic and transesophageal) and electronic health records (EHR) were queried for all patients aged ≥ 18 years who had at least one study during calendar years 2013 to 2018. All studies for any encounter diagnosis, inpatient and outpatient, trans-thoracic or trans-esophageal or stress echocardiography, complete and focused were retrieved, yielding 146,876 studies obtained on 48,524 unique patients.

The Institutional Review Board at Yale University approved this study. All methods were performed in accordance with the relevant guidelines and regulations.

### Analytic cohort building

This analysis included patients aged 40–95 years.

**Figure 1:**
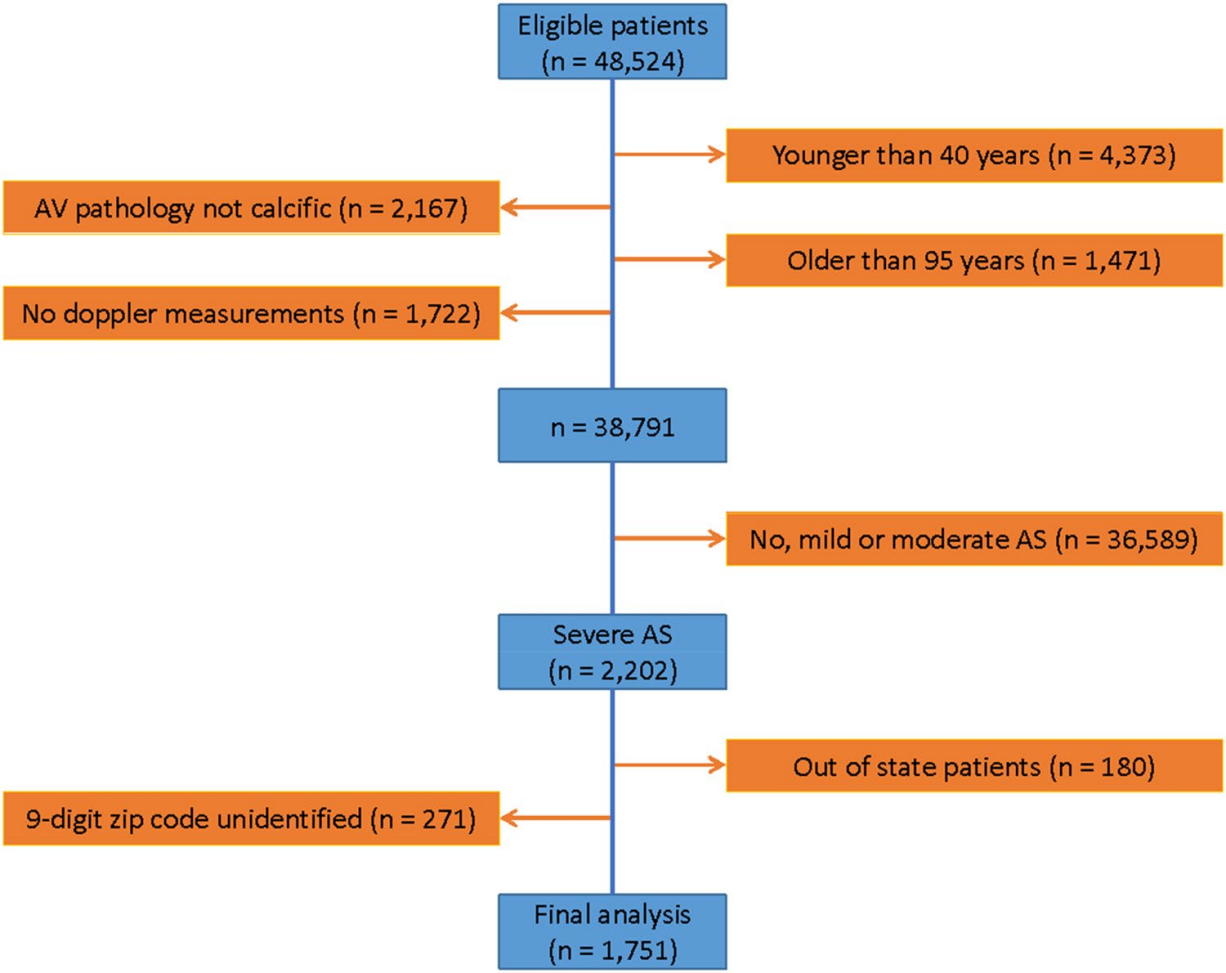
Diagram displaying patient inclusion and the cohort of study.

Using the echocardiography report and International Classification of Disease (ICD-10) codes, patients with the following conditions were excluded from analysis (Fig. 1):

1. prosthetic AV on initial echocardiography during the study period.
2. AV pathologies other than calcific AS (rheumatic, endocarditis, hypertrophic cardiomyopathy, moderate and severe AI, and aortic valve tumor), given their relative low incidence and to get a more homogenous cohort.
3. history of AVR as part of prior aortic aneurysm or dissection repair.
4. recipient of heart transplant or ventricular assist device.
5. echocardiography studies with no parameters to assess the degree of AS.

### Area deprivation index

The full address of residence of the patients included in the final cohort was used to identify the nine-digit zip code by searching the USPS website (https://tools.usps.com/zip-code-lookup.htm?byaddress). The ADI was identified by matching the patients nine-digit zip codes to the ADI scores as reported in the neighborhood atlas developed by the University of Wisconsin^16^.

Patients were divided into 5 ADI groups (A to E) based on their Connecticut State ADI rankings, each group containing two deciles, in ascending order, with Group A (rankings 1–2) being the least deprived, and Group E (rankings 9–10) being the most disadvantaged.

### Severity of aortic stenosis

According to Recommendations of the European Association of Cardiovascular Imaging and the American Society of Echocardiography^17^, AS was categorized as severe if aortic valve area (AVA) was ≤ 1 cm^2^, and/or dimensionless valve index (DVI) ≤ 0.25, or the maximum flow velocity across the valve (V-max) ≥ 4 m/sec, or mean pressure gradient across the valve (PG-mean) ≥ 40 mmHg. The first echocardiography during the study period that defined severe AS was chosen as the index study.

### Patient characteristics

Age was documented at the date of the index echocardiography examination. Race was categorized into Caucasian, African American (AA), and other races (Asian, Indian American and Alaskan American). Baseline body mass index was used. Smoking was defined by more than 5 years of smoking. Hypertension, diabetes mellitus, dyslipidemia, heart failure, chronic kidney disease, coronary heart disease, chronic lung disease, cerebral infarction, intracranial hemorrhage, peripheral vascular disease, atrial fibrillation, heart block, chronic liver disease/cirrhosis, pulmonary hypertension, history of cardiac arrest, dementia, obesity, malnutrition, frailty, inability to walk, and depression were chosen as commonly evaluated comorbidities in this age group and were defined using ICD-10 codes (Online Table 1). Arrythmias are reported in this study under disease presentation as one of the indications for echocardiographic assessment. Procedural history included: history of coronary artery bypass graft (CABG), percutaneous coronary intervention (PCI), cardiac pacemaker, and cardiac defibrillator.

### Disease presentation

The disease presentation was evaluated by text mining of the indication for echocardiography and sorted into murmur, dyspnea, chest pain, myocardial infarction (MI), HF, syncope, stroke, sepsis, arrhythmia, known AS, shock and respiratory failure (Supplementary Table 1).

### Cardiac structure and function

The echocardiography reports were searched by text mining for permutations of the commonly evaluated structures and functions during echocardiographic exam, which included: left ventricular (LV) hypertrophy, LV diastolic dysfunction, LV systolic dysfunction, LV ejection fraction (EF), right ventricular dysfunction, bicuspid aortic valve, left atrial dilatation, mitral regurgitation, and tricuspid regurgitation (Supplementary Table 1).

### Study outcomes and follow-up

The primary outcomes were intervention rates with AVR (SAVR and TAVR) and mortality rates according to the ADI group. The patients were followed up till one of two outcomes/end points happened: intervention or death. Both outcomes/endpoints could have happened anytime during the study period. Once patient experienced either outcome, follow up ends at this point. So, if the patient had intervention 30 days after the ECHO, follow up ends at 30 days as he/she reached the first end point. If the patient did not have intervention but died during the study period 200 days after the ECHO, follow up ends at 200 days as he/she experienced the second endpoint. If neither happened during the study period, patient was considered censored on the day when death data were extracted (01/23/2020).

Secondary outcomes included: age at presentation, mode of presentation, comorbidity profiles and gender and racial differences in each ADI group.

### Aortic valve intervention

Using unique identifiers (medical record number, last name, first name, and date of birth), patients with severe AS were linked to our institutional Society of Thoracic Surgery (STS) to capture all AVR procedures performed for this cohort either simple AVR or AVR combined with other procedures. The same method was used to capture all TAVR procedures in our TVT registry database.

To identify patients who possibly had an intervention not captured in both databases or performed at a different institution, specific ICD-10 (Z95.1 and Z95.2) were used to identify patients with history of AVR. The records of those patients were further reviewed for the date of intervention (Supplementary Table 2).

### Mortality data

Death dates were extracted from the Connecticut State Vital Statistics database by linking the patient first and last name and date of birth on the date of censoring, which was 01/23/2020.

### Statistical analysis

Categorical variables were expressed as counts and percentages, and continuous variables were presented as means and standard deviations or medians and interquartile range. Differences among the study groups were compared using Kruskal–Wallis test for continuous variables and Cochran-Armitage test for categorical variables.

The rates of surgical or transcatheter AVR (SAVR or TAVR) were compared among 5 ADI groups using competing risks analysis, which takes death as a competing event. The cumulative incidence function (CIF) was compared among 5 ADI ranking groups using the Fine and Gray method. The estimates of the sub-distributional hazard ratios of the outcome of intervention for multiple variables were calculated.

Data analysis was conducted using SAS 9.4 (SAS Institute Inc, Cary, NC). A two-sided *P* value of < 0.05 was considered statistically significant.

### Conference presentation

The study was presented for discussion at the 103rd Annual Meeting of American Association for Thoracic Surgery.

### Institutional review board statement

The Yale University Human Investigation Committee (Institutional Review Board at Yale University) approved this study. IRB protocol ID: 2000028791 Approval date: 9/2/2020.

### Informed consent

The study was approved by Yale University Human Investigation Committee, the Institutional Review Board at Yale University, IRB Protocol ID 2000028791, Approval Date 9/2/2020. Informed consent was waived by the Yale University Human Investigation Committee due to the retrospective chart review nature of the study.

## Results

The final analytic cohort included 1,751 patients with severe AS who were residents of the State of Connecticut at the time of their echocardiography and had an identifiable non-institutional residential nine-digit zip code (Fig. 1).

For the whole cohort, the median duration of follow-up and inter-quartile range were 2.8 (1.52–4.8) years.

### Baseline characteristics

As shown in Table 1 listing data for Groups A to E, the more deprived ADI groups tended to be younger (79.0 ± 9.3 vs 79.0 ± 9.5 vs 79.3 ± 10.1 vs 77.9 ± 11.9 vs 75.4 ± 12.4 years, *P* = 0.002), even when stratified into multiple age groups as in Supplementary Table 3. The more deprived ADI groups were also more likely to be female (39.9% vs 44.2% vs 50.0% vs 50.8% vs 55.3%, *P* < 0.001), African American (0.7% vs 1.6% vs 3.7% vs 7.0% vs 23.0%), other minority races (3.3% vs 4.8% vs 3.2% vs 6.6% vs 10.6%, *P* < 0.001), and were more likely to have a history of stroke (12.0% vs 11.8% vs 11.5% vs 13.9% vs 18.0%, *P* = 0.032), chronic lung disease (19.6% vs 22.1% vs 25.2% vs 26.3% vs 27.5%, *P* = 0.010), diabetes mellitus (27.2% vs 26.6% vs 31.9% vs 35.6% vs 42.6%, *P* < 0.001), and obesity (6.0% vs 5.2% vs 5.7% vs 6.3% vs 11.1%, *P* = 0.002), while the lower ADI groups tended to have a history of atrial fibrillation (35.9% vs 36.0% vs 36.0% vs 33.5% vs 26.6%, *P* < 0.026) and dyslipidemia (*P* = 0.012).

**Table 1:** Demographics and comorbidities.

Variable	Group A ADI = 1, 2 (n = 301, %)	Group B ADI = 3, 4 (n = 439, %)	Group C ADI = 5, 6 (n = 436, %)	Group D ADI = 7, 8 (n = 331, %)	Group E ADI = 9, 10 (n = 244, %)	*P* value
Demographics						
Age, mean	79.0 ± 9.3	79.0 ± 9.5	79.3 ± 10.1	77.9 ± 11.9	75.4 ± 12.4	**0.002**
Sex, female	120 (39.9)	194 (44.2)	218 (50.0)	168 (50.8)	135 (55.3)	**< 0.001**
Race						**< 0.001**
African American	2 (0.7)	7 (1.6)	16 (3.7)	23 (7.0)	56 (23.0)	
Caucasian	289 (96)	411 (93.6)	406 (93.1)	286 (86.4)	162 (66.4)	
Other races	10 (3.3)	21 (4.8)	14 (3.2)	22 (6.6)	26 (10.6)	
Body mass index, kg/m^2^	27.2 ± 6.0	27.8 ± 6.7	28.1 ± 6.9	28.8 ± 7.2	28.4 ± 7.5	0.138
Comorbidities						
Smoking	76 (25.2)	131 (29.8)	133 (30.5)	96 (29.0)	62 (25.4)	0.995
Hypertension	232 (77.1)	345 (78.6)	353 (81.0)	260 (78.5)	198 (81.1)	0.294
Coronary artery disease	136 (45.2)	200 (45.6)	208 (47.7)	144 (43.5)	108 (44.3)	0.681
Dyslipidemia	199 (66.1)	268 (61.1)	293 (67.2)	189 (57.1)	137 (56.1)	**0.013**
Pulmonary hypertension	14 (4.6)	15 (3.4)	19 (4.4)	17 (5.1)	7 (2.9)	0.789
Heart Block	27 (9.0)	36 (8.2)	44 (10.1)	28 (8.5)	20 (8.2)	0.878
Atrial fibrillation	108 (35.9)	158 (36.0)	157 (36.0)	111 (33.5)	65 (26.6)	**0.026**
Stroke	36 (12.0)	52 (11.8)	50 (11.5)	46 (13.9)	44 (18.0)	**0.032**
PVD	33 (11.0)	48 (10.9)	60 (13.8)	42 (12.7)	21 (8.6)	0.811
Chronic lung disease	59 (19.6)	97 (22.1)	110 (25.2)	87 (26.3)	67 (27.5)	**0.010**
Chronic kidney disease	44 (14.6)	70 (15.9)	83 (19)	58 (17.5)	41 (16.8)	0.335
Diabetes mellitus	81 (27.2)	117 (26.6)	139 (31.9)	118 (35.6)	104 (42.6)	** < 0.001**
Chronic liver disease	9 (3.0)	16 (3.6)	12 (2.7)	12 (3.6)	10 (4.1)	0.574
Dementia	12 (4.0)	23 (5.2)	28 (6.4)	25 (7.5)	13 (5.3)	0.182
Depression	25 (8.3)	49 (11.2)	54 (12.4)	44 (13.3)	26 (10.7)	0.197
Obesity	18 (6.0)	23 (5.2)	25 (5.7)	21 (6.3)	27 (11.1)	**0.002**
Heart failure	66 (21.9)	87 (19.8)	100 (22.9)	71 (21.4)	60 (24.6)	0.355

Significant values are in [bold].

Values are expressed as mean ± standard deviation or n (%).

*ADI* Area deprivation index, *PVD* Peripheral vascular disease.

### Disease presentation

The more disadvantaged groups were more likely to present with sepsis (0.3% vs 1.8% vs 1.1% vs 1.5% vs 3.3%, *P* = 0.031) and various arrhythmias, including both atrial and ventricular arrhythmias, (2.0% vs 5.2% vs 4.4% vs 7.6% vs 5.3%, *P* = 0.022) to initiate workup for AS, while the less deprived groups were more likely to have a known diagnosis of AS (44.8% vs 39.4% vs 31.5% vs 29.9% vs 27.0, *P* < 0.001) prior to presentation (Table 2).

**Table 2:** Presentation, intervention and mortality.

Variable	Group A ADI = 1, 2 (n = 301, %)	Group B ADI = 3, 4 (n = 439, %)	Group C ADI = 5, 6 (n = 436, %)	Group D ADI = 7, 8 (n = 331, %)	Group E ADI = 9, 10 (n = 244, %)	*P* value
Presentation						
Murmur	13 (4.3)	19 (4.3)	17 (3.9)	17 (5.1)	19 (7.8)	0.076
Dyspnea	21 (7)	41 (9.3)	30 (6.9)	27 (8.2)	25 (10.2)	0.420
Chest pain	7 (2.3)	13 (3.0)	20 (4.6)	9 (2.7)	10 (4.1)	0.337
Heart failure	23 (7.7)	67 (15.3)	62 (14.2)	49 (14.8)	32 (13.1)	0.113
MI	5 (1.7)	18 (4.1)	21 (4.8)	16 (4.8)	5 (2.1)	0.550
Syncope	8 (2.7)	19 (4.3)	14 (3.2)	14 (4.2)	12 (4.9)	0.273
Stroke	12 (4.0)	9 (2.1)	13 (3.0)	7 (2.1)	15 (6.1)	0.248
Sepsis	1 (0.3)	8 (1.8)	5 (1.1)	5 (1.5)	8 (3.3)	**0.031**
Arrhythmia	6 (2.0)	23 (5.2)	19 (4.4)	25 (7.6)	13 (5.3)	**0.022**
Known AS	134 (44.8)	173 (39.4)	137 (31.5)	99 (29.9)	66 (27.0)	** < 0.001**
Shock	9 (3.0)	8 (1.8)	5 (1.1)	17 (5.1)	5 (2.0)	0.443
Respiratory failure	1 (0.3)	6 (1.4)	2 (0.5)	3 (0.9)	1 (0.4)	0.776
Intervention						
Pacemaker	17 (5.6)	31 (7.0)	28 (6.4)	17 (5.1)	14 (5.7)	0.619
Defibrillator	7 (2.3)	16 (3.6)	15 (3.4)	8 (2.4)	2 (0.8)	0.177
CABG	59 (19.6)	84 (19.1)	83 (19.0)	41 (12.4)	26 (10.7)	** < 0.001**
PCI	48 (16)	63 (14.3)	60 (13.8)	42 (12.7)	29 (11.9)	0.129
AVR	158 (52.5)	206 (46.9)	201 (46.1)	162 (48.9)	97 (39.7)	**0.023**
Mortality	104 (34.5)	177 (40.3)	165 (37.8)	127 (38.4)	86 (35.2)	0.925

Significant values are in [bold].

Values are expressed as n (%).

*ADI* Area deprivation index, *MI* Myocardial infarction, *CABG* Coronary artery bypass graft, *PCI* Percutaneous coronary intervention, *AVR* Aortic valve replacement.

### Cardiac structure and function

Although the more deprived ADI groups were likely to have severe RV dysfunction (0.3% vs 1.4% vs 0.2% vs 0.9% vs 3.3%, *P* = 0.017), Doppler parameters (including AVA, DVI, V-max, and PG-mean), LVEF, and other cardiac structural changes did not differ significantly among the groups (All *P* > 0.05) (Table 3).

**Table 3:** Echocardiographic measurements.

Variable	Group A ADI = 1, 2 (n = 301, %)	Group B ADI = 3, 4 (n = 439, %)	Group C ADI = 5, 6 (n = 436, %)	Group D ADI = 7, 8 (n = 331, %)	Group E ADI = 9, 10 (n = 244, %)	*P* value
AVA, cm^2^	0.8 ± 0.2	0.8 ± 0.2	0.8 ± 0.2	0.8 ± 0.2	0.8 ± 0.2	0.159
V_max_, m/s	3.9 ± 0.8	4.7 ± 0.9	3.9 ± 0.8	3.7 ± 0.8	3.7 ± 0.9	0.160
PG, mmHg	39.7 ± 15.4	33.5 ± 15.3	35.5 ± 14.9	34.2 ± 14.0	34.4 ± 16.3	0.193
DVI	0.3 ± 0.1	0.3 ± 0.1	0.3 ± 0.1	0.3 ± 0.1	0.4 ± 0.1	0.970
LVEF, %	58.4 ± 13.4	57.1 ± 14	57.2 ± 13.8	56.4 ± 14.9	56.4 ± 14.9	0.432
LV systolic dysfunction						
Mild	24 (8.0)	38 (8.7)	33 (7.6)	27 (8.2)	27 (11.1)	0.357
Moderate	13 (4.3)	18 (4.1)	39 (8.9)	18 (5.4)	13 (5.3)	0.310
Severe	15 (5.0)	26 (5.9)	18 (4.1)	19 (5.7)	8 (3.3)	0.387
LV hypertrophy						
Mild	125 (41.5)	170 (38.7)	182 (41.7)	132 (39.9)	105 (43.0)	0.637
Moderate	31 (10.3)	45 (10.2)	50 (11.5)	40 (12.1)	26 (10.7)	0.580
Severe	3 (1.0)	5 (1.1)	5 (1.1)	5 (1.5)	4 (1.6)	0.426
RV dysfunction						
Mild	36 (12.0)	63 (14.3)	55 (12.6)	46 (13.9)	23 (9.4)	0.414
Moderate	13 (4.3)	22 (5.0)	24 (5.5)	24 (7.2)	10 (4.1)	0.520
Severe	1 (0.3)	6 (1.4)	1 (0.2)	3 (0.9)	8 (3.3)	**0.017**
LA dilation						
Mild	37 (12.3)	59 (13.4)	66 (3.8)	41 (12.4)	37 (15.2)	0.504
Moderate	27 (9.0)	62 (14.1)	51 (11.7)	45 (13.6)	25 (10.2)	0.749
Severe	71 (23.6)	115 (26.2)	107 (24.5)	87 (26.3)	49 (20.1)	0.449
Mitral regurgitation						
Mild	84 (27.9)	126 (28.7)	109 (25.0)	83 (25.1)	61 (25.0)	0.204
Moderate	22 (7.3)	50 (11.4)	50 (11.5)	30 (9.1)	32 (13.1)	0.159
Severe	14 (4.6)	13 (3.0)	13 (3.0)	9 (2.7)	7 (2.9)	0.252
Tricuspid regurgitation						
Mild	52 (17.3)	78 (17.8)	82 (18.8)	65 (19.6)	51 (20.9)	0.207
Moderate	38 (12.6)	52 (11.8)	58 (13.3)	47 (14.2)	30 (12.3)	0.677
Severe	11 (3.6)	28 (6.4)	14 (3.2)	14 (4.2)	9 (3.7)	0.448
Bicuspid aortic valve	7 (2.3)	8 (1.8)	6 (1.4)	3 (0.9)	8 (3.3)	0.884
AAo aneurysm	33 (11.0)	50 (11.4)	42 (9.6)	35 (11.0)	36 (14.7)	0.352

Significant values are in [bold].

Values are expressed as mean ± standard deviation or n (%).

*LV* Left ventricular, *EF* Ejection fraction, *PG* Pressure gradient, *RV* Right ventricular, *AVA* Aortic valve area, *V*_*max*_ Maximum flow velocity, *PG* Pressure gradient, *DVI* Dimensionless valve index, *AAo* Ascending aortic aneurysm.

### Aortic valve replacement (AVR or TAVR)

The more disadvantaged ADI groups were less likely to undergo AVR (52.5% vs 46.9% vs 46.1% vs 48.9% vs 39.7%, *P* = 0.023) and CABG (Table 2).

Amongst patients who had intervention, there was no statistically significant difference in the mode of intervention or the time to intervention. ADI group E had the highest proportion of surgical AVR (40.5% vs 34.5% vs 35.3% vs 33.9% vs 48.4%, *P* = 0.4986) and the longest time to intervention (215.1 ± 349.9 vs 268 ± 340.7 vs 239.2 ± 323.8 vs 267.8 ± 386.8 vs 280.9 ± 408.1, *P* = 0.1285) in days. Yet, both results were not statistically different.

### Mortality

The unadjusted overall mortality rates showed no significant difference among 5 ADI groups (34.5% vs 40.3% vs 37.8% vs 38.4% vs 35.3%, *P* = 0.925) (Table 2).

### Competing risk analysis of aortic valve intervention

**Figure 2:**
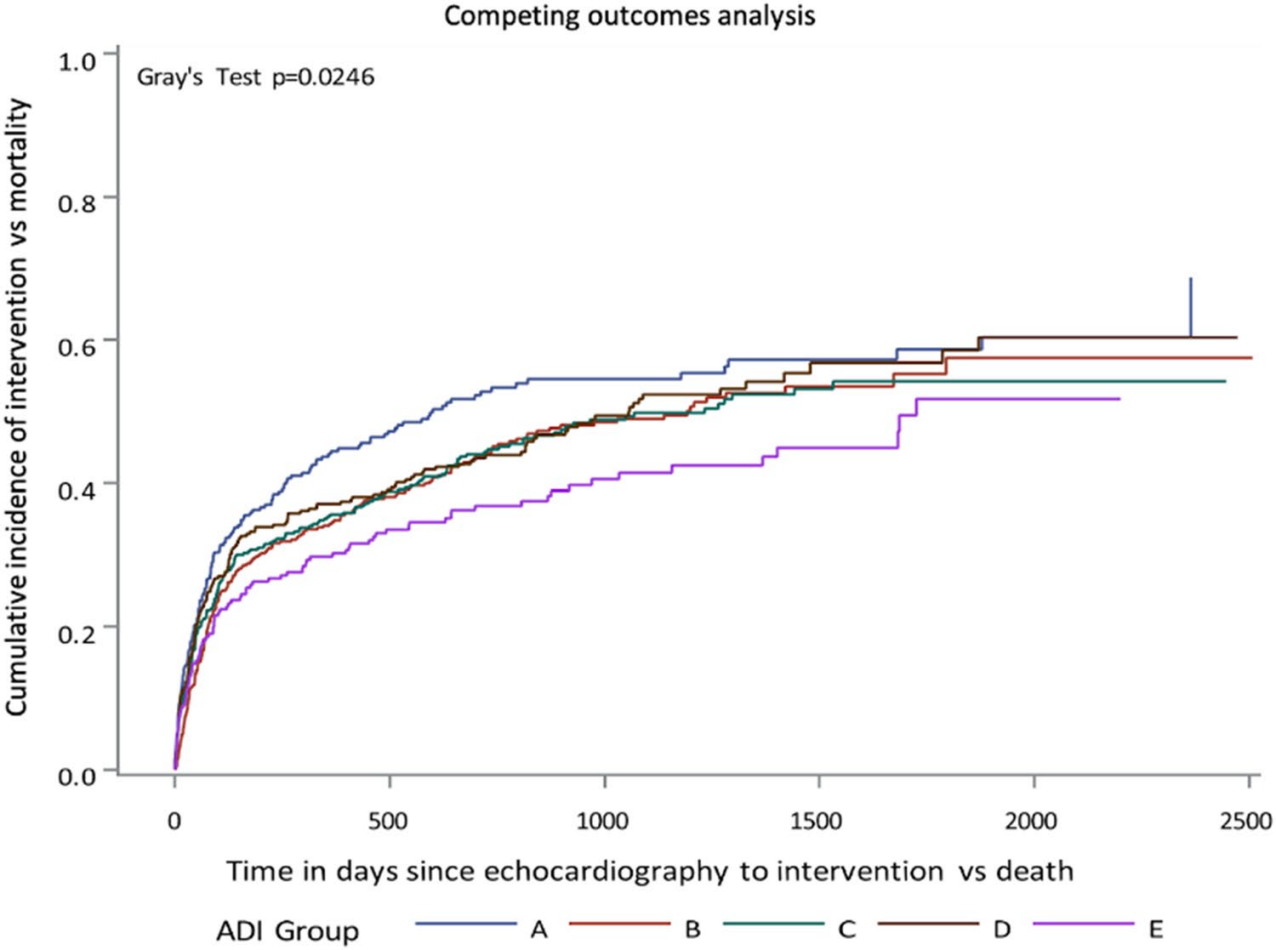
Competing risks analysis shows that the rates of undergoing aortic valve replacement for severe aortic stenosis differed significantly among patients of 5 groups of area deprivation index (ADI) rankings, which was highest in Group A and lowest in Group E.

**Figure 3:**
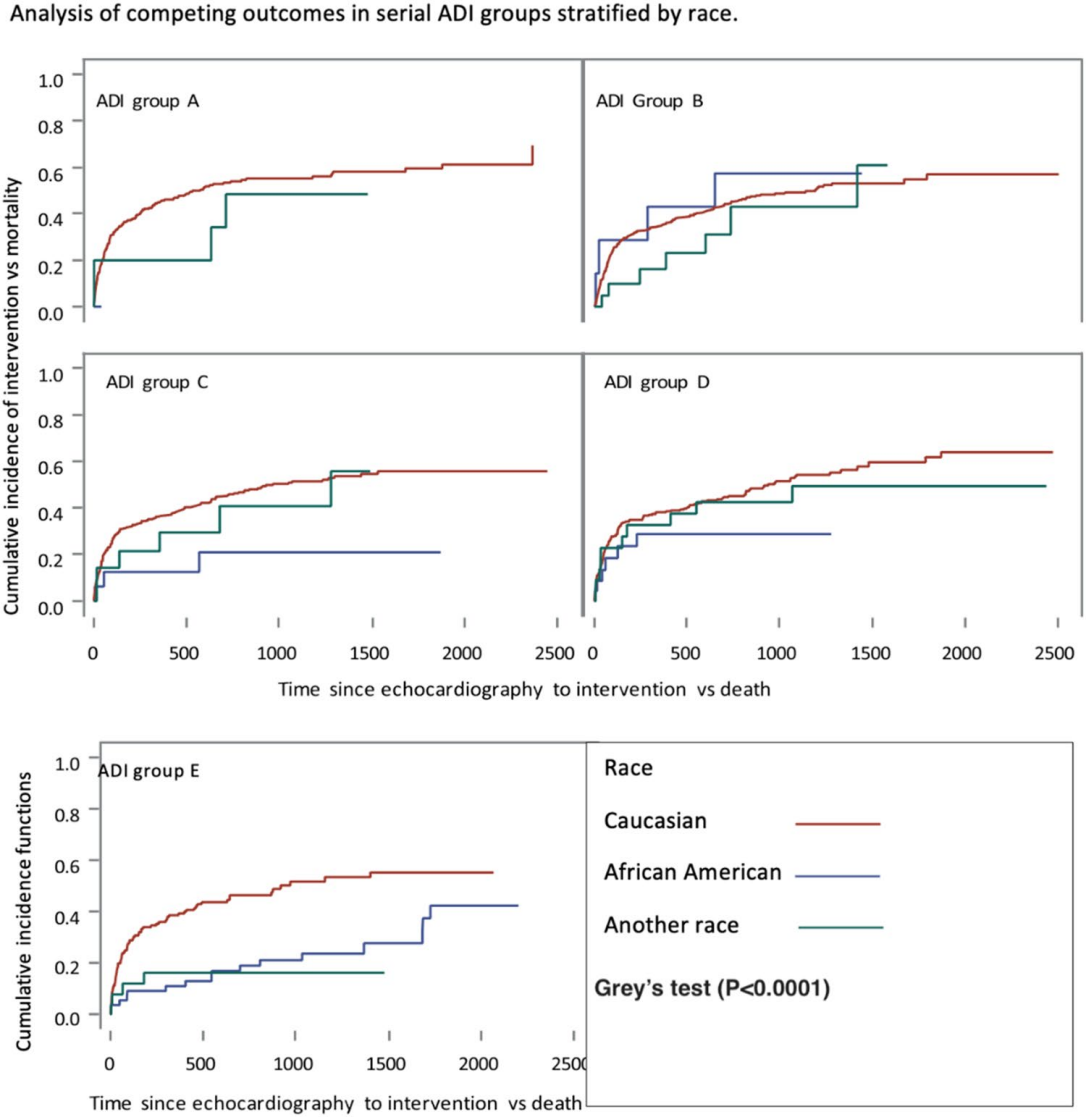
Group analysis showed significant association between race and aortic valve intervention.

**Figure 4:**
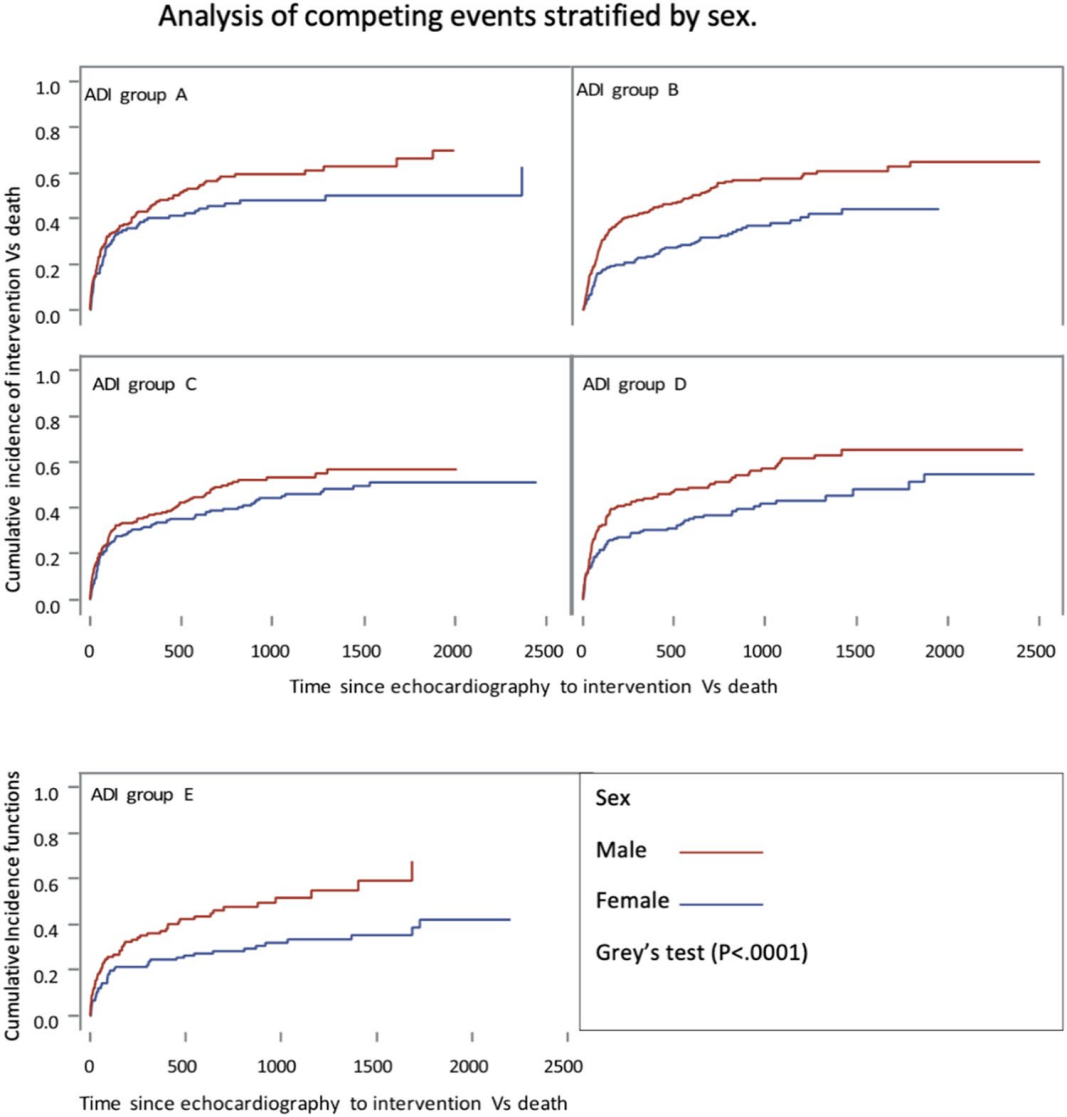
Group analysis showed significant association between sex and aortic valve intervention.

The CIF function of the Fine and Gray method showed that patients in Group E (the most disadvantaged) were the least likely to undergo AV intervention, patients in Group A (the least disadvantaged) were the most (Gray’s test, *P* = 0.025) (Fig. 2). The association between ADI ranking and intervention rates was significantly influenced by sex and race. In within group analysis, there was significant association between race and intervention (Gray’s test, *P* < 0.001) (Fig. 3), and between sex and intervention (Gray’s test, *P* < 0.001) (Fig. 4). When adjusted for other factors, the competing risk analysis model showed increasing age (hazard ratio [HR] = 0.991; 95% confidence interval [CI] 0.984–0.998, *P* < 0.001), female gender (HR = 0.654; 95% CI 0.568–0.755, *P* < 0.001), African American race (HR = 0.459; 95% CI 0.311–0.679, *P* < 0.001), and other minority race (HR = 0.516; 95% CI 0.359–0.743, *P* < 0.001) predicted less likelihood of undergoing AVR. However, ADI ranking, EF, and types of insurance were not statistically significant (Table 4).

**Table 4:** Multivariable analysis of the factors associated with aortic valve intervention in patients with severe aortic stenosis when death is a competing event.

Candidate variable	Hazard ratio	95% confidence interval	*P* value
ADI ranking (group A as reference)			
Group B	0.858	0.697–1.056	0.148
Group C	0.894	0.725–1.102	0.294
Group D	0.929	0.744–1.160	0.515
Group E	0.870	0.666–1.136	0.305
Age, year	0.991	0.984–0.998	**0.007**
Female gender	0.654	0.568–0.755	** < 0.001**
Race			
African American	0.459	0.311–0.679	**< 0.001**
Other	0.516	0.359–0.743	** < 0.001**
Ejection fraction %	1.002	0.996–1.007	0.543
Insurance			
Commercial	1.150	0.933–1.418	0.277
Self pay	1.207	0.763–1.910	0.651

Significant values are in [bold].

*ADI* Area deprivation index.

## Discussion

The longitudinal nature of this study enabled us to explore different issues in the care of patients with aortic stenosis according to neighborhood disadvantage. We found that the ADI rank was associated with mortality and intervention rates in patients with severe AS. Adjusted for other factors, the difference in outcomes among ADI groups was found to interact with age, gender, and race.

Patients from more deprived neighborhoods were more likely to be females and of African American and other races. Living in such deprived environments leads to a higher burden of traditional cardiovascular risk factors in addition to behavioral, socioeconomic, and psychological factors that predispose and accentuate their risk of poor cardiovascular health^18^. Although it is generally believed that the fibrocalcific nature of AS means that valve calcification rises dramatically with age, particularly at 65 years^19^, the fact that AS is a result from valvular calcification implies that with common cardiovascular risk factors, the development of AS can be similar to other diseases like CAD and PVD. The higher burden of traditional cardiovascular risk factors in these groups was evident by the association of increasing ADI with increased rates of obesity, DM, stroke, and chronic lung disease in this study. This may explain, at least in part, the higher disease burden of AS in distressed neighborhoods.

Also, patients from more deprived neighborhoods were less likely to have a previous diagnosis of AS. In this study, only 29% of patients in ADI group 5 had known AS before diagnosis with severe AS. In a study from Sweden, a cohort of 1586 subjects screened with echocardiography from different socioeconomic status (SES), the prevalence of AS was 2.5% in high SES vs 3.9% in moderate SES and 6.4% in low SES (*P* = 0.05). The study also showed that the prevalence of undiagnosed AS was 3.1%^20^. This means that the prevalence of undiagnosed AS in deprived populations is high, which is a very concerning issue in the care of such patients. As a potential solution, we have developed a screening algorithm for moderate and severe AS^21^, and encourage clinicians to use it for early identification of this disease.

Although AS is a disease primarily affecting the elderly, advanced age has been a barrier to aortic valve intervention, especially SAVR^1^. TAVR was developed as an alternative to SAVR for patients of advanced age with severe AS and has achieved favorable outcomes in octogenarians and nonagenarians^22^. In this study, we refined the age criteria to 40–95 years, which we believe is the age group for whom AVR could increase life expectancy in relatively younger patients and improve quality of life in older patients. This age criteria were chosen based on multiple considerations. First, as conceptualized by Ross and Braunwald (1968) and confirmed in numerous studies^5^, symptomatic demarcation occurs in middle age (40 years), most patients develop symptoms in the 7th through 9th decade. Second, exclusion of patients younger than 40 years helped decrease the number of outliers that may affect study outcomes if they had different etiologies like rheumatic AS, and the amount of work during data extraction. Lastly, the oldest patient undergoing intervention in this cohort was aged 95 years at the time of TAVR. Theoretically any patient younger than this age could be eligible for intervention and hence the age 95 was chosen as an upper limit. In a recent study by Pesarini and associates^23^, TAVR was associated with improved quality of life in both patients with and without sarcopenia, a marker of frailty, which implies that it is reasonable to offer intervention to the elderly population. Yet, the overall percentage of patients undergoing intervention in this study is low (47%) and the nadir was in the most disadvantaged groups which are relatively younger and could experience increase life expectancy after AVR. Further research is needed to address this import gap in management of severe AS.

Even though women with severe AS are more likely to develop symptoms compared to men^24^, multiple studies consistently report lower rates of referral^25^ and lower rates of intervention in women^26^. Recent reports show similar representation of men and women in TAVR registries^27–30^. The improved mortality of women after TAVR compared to SAVR might explain the pattern and help close the gap in the care of women with severe AS. This also highlights prevalent bias in the healthcare system regardless of ADI status.

Patients of African American and other minority races were less likely to undergo AVR in this study, which is consistent with previous reports that show African Americans are likely to refuse SAVR^31,32^ or less likely to be referred to specialists^32^. Studies show that TAVR did not close this gap in patient care as African American patients underwent TAVR less often compared to Caucasian patients^33–35^. Once again, we see that African-Americans, particularly female, irrespective of their ADI status, experience bias in referral rates and thus have decreased access to life improving or life-extending measures.

The results of this study suggest that gaps emphasizing inequality still exist in the care of elderly patients, women, and minorities with severe aortic stenosis. Interestingly, the within ADI group analysis showed that gender and race significantly impacted AVR intervention rates, suggesting that these factors may contribute to clinical gap regardless of the ADI status of the patient. Possibly, special attention may need to be tailored by health systems to ensure that the clinical gap accentuated by factors such as gender and race is minimized in the local communities they serve. Remedial measures can include creating a community screening echocardiogram program in areas with higher ADI, alerts implemented into the electronic medical record to screen patients with risk factors, and crafting an implicit bias workshop to highlight these existing disparities and to challenge providers to address these inequities. Careful assessment of the risks and benefits of AVR in the elderly may improve clinical decision making, which entails weighing the possibility of repeated admissions for HF, poor quality of life, and high mortality risk against the morbidity and mortality of AVR in such patients. We recently developed a risk prediction model to calculate 1- and 5-year mortality risk in AS patients with and without intervention, accounting for patient demographics and comorbidities^36^. Disparities in health care of AS patients based on gender and race are complex and multifactorial. The National Heart, Lung, and Blood Institute Working Group in a special report proposed tools such as an echo alert that could be deployed within health care systems and may enhance the equity of care (timely referral and treatment especially if intervention is indicated) regardless of gender or race^37^. Institution of such simple but powerful clinical alerts across a large health system such as the one studied in this report may help bridge the inequity in the care of patients with AS based on socioeconomic factors.

### Study limitations

This study has several limitations. Most important of all, this is a single center study, and the results may not be generalizable to other states in US. However, there are no nationwide databases that could address the question of the study with sufficient granular data. Previous studies from the STS/TAVR database or data from the Medicare/Medicaid centers were limited to studying the difference in survival after the intervention. In this sense, our study provides information on the course of the disease before the intervention and points to areas of deficiencies in the healthcare system that could be improved upon. The retrospective nature of this study does not allow for precise judgment of the eligibility for intervention in this cohort, although most of the patients with severe AS were symptomatic as documented in the indication for the echocardiography. The tertiary care setting of our institution may have biased the cohort towards patients with higher mortality risk, despite adjustment of multiple factors that were made in the multivariable analyses. Another important limitation of the ADI is that it assigns the same index to all individuals living in the same census-block group. This may lead to misclassification of some deprived patients with more advantaged neighborhoods e.g. undocumented immigrants and poor families living at the periphery of rich neighborhoods. Yet, we believe that the effect of this phenomenon led to narrowing the difference in outcomes between the groups and if accounted for, would have led to a more pronounced difference in outcomes, which aligns with the results of the study. Data on the cause of death in this cohort was not available and it is possible that some patients died from non-cardiovascular causes. The question whether AVR prevents non-cardiovascular death or not is not well investigated so far and warrants further research.

## Conclusions

The results of this study show that patients with severe AS living in more deprived neighborhoods were less likely to undergo aortic valve interventions, which was influenced by female gender, and African American race. Continued efforts are needed to improve equity in the care of patients with severe aortic stenosis from distressed neighborhoods, particularly females and those of African American and other minority races.

### Supplementary Information

Supplementary Information.

## Data Availability

The datasets generated and/or analyzed during the current study are not publicly available due to the sensitive nature of the data used in this study but are available from the corresponding author on reasonable request.
