# Supplementary material for: Living in disadvantaged neighborhoods linked to less intervention for severe aortic stenosis
Source: Sci Rep. 2024 Feb 28;14:4952. doi: 10.1038/s41598-024-52660-w (PMC10902341; doi:10.1038/s41598-024-52660-w)
Supplement: Supplementary file 1 — Supplementary Information. [file 41598_2024_52660_MOESM1_ESM.docx]

**SUPPLEMENTARY TABLES**

**Supplementary Table 1. Text permutations used in text mining.**

| **Parameter** | **Permutations** |
| --- | --- |
| Left ventricular systolic function | “Mildly decreased left ventricular systolic function”, “mild decrease systolic function”, “mildly decreased systolic, function”, “Moderately decreased systolic function”, “Moderately decreased left ventricular systolic function”, “Severely decreased systolic function”, “Severely decreased left ventricular systolic function”, “The left ventricular systolic function is now severely decreased” |
| Right ventricular function | “Mildly reduced right ventricular systolic function”, “Mildly decreased right ventricular systolic function”, “RIGHT VENTRICLE:, Normal right ventricular size. Mildly decreased systolic function”, “Severely increased right ventricular size with moderately decreased, systolic function”, “Severely increased right ventricular size. Moderately decreased, systolic function”, “RIGHT VENTRICLE:, Severely increased right ventricular size. Moderately decreased, systolic function”, “Moderately decreased right ventricular systolic function”, “The right ventricular function is mildly decreased”, “Severely decreased right ventricular systolic function” |
| Diastolic dysfunction | “Mild diastolic dysfunction”, “Mild diastolic, dysfunction”, “Mild , diastolic dysfunction”, “Mild diastolic , dysfunction”, “Moderate diastolic dysfunction”, “Moderate , diastolic dysfunction”, ”Moderate , diastolic dysfunction”, “Moderate , diastolic dysfunction”, “Severe diastolic dysfunction”, “Severe , diastolic dysfunction” |
| Left ventricular hypertrophy | “Mild concentric hypertrophy”, “Mild concentric , hypertrophy”, “Mild left , ventricular concentric hypertrophy”, “Moderate concentric , hypertrophy”, “Moderate concentric left ventricular hypertrophy”, “Mild concentric left ventricular hypertrophy”, “Mild concentric ,left ventricular hypertrophy”, “Mild concentric left ,ventricular hypertrophy”, “Mild concentric left, ventricular hypertrophy”, “Mild concentric ,left ventricular hypertrophy”, “Mild concentric left, ventricular hypertrophy”, “Mild concentric left ,ventricular hypertrophy”, “Mild left ventricular hypertrophy”, “Severe concentric hypertrophy”, “Severe concentric , hypertrophy”, “Severe left , ventricular concentric hypertrophy”, “Severe concentric , hypertrophy”, “Severe concentric left ventricular hypertrophy”, “Severe concentric left ventricular hypertrophy”, “Severe concentric ,left ventricular hypertrophy”, “Severe concentric left ,ventricular hypertrophy”, “Severe concentric left, ventricular hypertrophy”, “Severe concentric ,left ventricular hypertrophy”, “Severe concentric left, ventricular hypertrophy”, “Severe concentric left ,ventricular hypertrophy”, “Severe left ventricular hypertrophy” |
| Left atrial dilatation | “Mild left atrial dilatation”, “moderate left atrial dilatation”, “severe left atrial dilatation”, “Severe biatrial enlargement”, “mild biatrial enlargement”, “left atrium measures to be, severely dilated”, “Left atrium is severely dilated”, “left atrium mildly dilated”, “left atrium is moderately dilated”, moderate biatrial enlargement”, “moderate biatrial dilatation” |
| Mitral regurgitation | “Mild mitral regurgitation”, “Moderate mitral regurgitation”, “Mild to moderate mitral regurgitation”, “moderate tricuspid and mitral regurgitation”, “Mild-moderate mitral regurgitation”, “Mild mitral and tricuspid valve regurgitation”, “moderate mitral valve regurgitation”, ”severe mitral regurgitation”, “moderate to severe mitral regurgitation”, “severe mitral valve regurgitation” |
| Tricuspid regurgitation | “Mild tricuspid regurgitation”, “mild tricuspid valve regurgitation”, “Moderate tricuspid regurgitation”, “moderate tricuspid and mitral regurgitation”, “Moderate - severe tricuspid regurgitation”, “Severe tricuspid regurgitation”, “severe tricuspid valve regurgitation” |
| Ascending  aortic aneurysm | “Dilated ascending aorta”, “dilated sinuses of valsalva”, “ascending aortic dilation”, “ascending aortic dilatation”, “ascending aortic aneurysm”, “aneurysmal ascending aorta”, “dilatation of the ascending thoracic aorta” |
| Bicuspid aortic valve | “bicuspid” |
| Murmur | “murmur”, “murmur” |
| Dyspnea | “dyspnea”, “sob”, “doe”, “shortness of breath” |
| Chest pain | “chest pain”, “cp”, “chest discomfort” |
| Heart failure | “heart failure”, “chf”, “bnp”, “odema”, “edema” |
| Myocardial infarction | “stemi”, “trop”, “acs”, “acute coronary syndrome” |
| Syncope | “syncope”, “fall” |
| Stroke | “stroke”, “tia” |
| Sepsis | “sept”, “sepsis”, “bacter” |
| Arrhythmias | “arrhythm”, “brady”, “fib”, “flutter”, “arrest”, “tach”, “PEA”, “block”, “chb” |
| Known AS | “AS”, “aortic stenosis”, “aortic valve stenosis”, severe AS”, “critical AS”, “AVR” |
| Shock | “shock”, “hypotension”, “low blood pressure”, “low bp”, “pressor” |
| Respiratory failure | “respiratory failure”, ”hypoxia”, “hypercarb”, “oxygen”, “o2”, “co2” |

**Supplementary Table 2. International classification of disease- version 10 (ICD-10) codes used to characterize comorbidities**

| **Hypertension** | **I10, I11, I11.0, I11.9, I12, I12.0, I12.9, I13, I13.0, I13.1, I13.10, I13.11, I13.2, I15, I15.0, I15.1, I15.2, I15.8, I15.9** |
| --- | --- |
| Coronary heart disease | I20, I20.0, I20.1, I20.8, I20.9, I21, I21.0, I21.01, I21.02, I21.09, I21.1, I21.11, I21.19, I21.2, I21.21, I21.29, I21.3, I21.4, I21.9, I24.8, I24.9, I25, I25.1, I25.10, I25.11, I25.11, I25.110, I25.111, I25.118, I25.119, I25.2, I25.3, I25.5, I25.6, I25.8, I25.81, I25.82, I25.83, I25.84, I25.89 |
| Dyslipidemia | E78, E78.0, E78.00, E78.01, E78.1, E78.2, E78.3, E78.4, E78.41, E78.49, E78.5, E78.6 |
| Pulmonary hypertension | I27.0, I27.2, I27.20, I27.21, I27.22, I27.23, I27.24, I27.29, I27.81 |
| Endocarditis | I33.0, I33.9, I38, I39 |
| Mitral regurgitation | I34.0, I34.1, I34.8, I34.9 |
| Aortic stenosis | I35.0, I35.2 |
| Tricuspid regurgitation | I36.1, I36.8, I36.9 |
| Cardiomyopathy | I42.0, I42.1, I42.2 |
| Heart block | I44.0, I44.1, I44.2, I44.30, I44.39, I44.4, I44.5, I44.60, I44.69, I44.7, I45.0, I45.10, I45.19, I45.2, I45.3, I45.4, I45.6, I45.81, I45.89, I45.9 |
| Cardiac arrest | I46.2, I46.8, I46.9 |
| Atrial fibrillation | I48.0, I48.1, I48.11, I48.19, I48.2, I48.20, I48.21, I48.91, I48.3, I48.4, I48.92, I49.5 |
| Heart failure | I11.0, I13.0, I13.1, I13.10, I13.11, I13.2, I50.1, I50.2, I50.20, I50.21, I50.22, I50.23, I50.3, I50.30, I50.31, I50.32, I50.33, I50.4, I50.40, I50.41, I50.42, I50.43, I50.8, I50.81, I50.810, I50.811, I50.812, I50.813, I50.814, I50.82, I50.84, I50.9 |
| Stroke | I60, I60.0, I60.00, I60.01, I60.02, I60.1, I60.10, I60.11, I60.12, I60.2, I60.3, I60.30, I60.31, I60.32, I60.4, I60.5, I60.50, I60.51, I60.52, I60.6, I60.6, I60.7, I60.8, I60.9, I61, I61.0, I61.1, I61.2, I61.3, I61.4, I61.5, I61.6, I61.8, I61.9, I62, I62.0, I62.00, I62.01, I62.02, I62.03, I62.1, I62.9, I63, I63.0, I63.00, I63.01, I63.011, I63.012, I63.013, I63.019, I63.03, I63.031, I63.032, I63.033, I63.039, I63.09, I63.1, I63.10, I63.11, I63.111, I63.112, I63.113, I63.119, I63.12, I63.13, I63.131, I63.132, I63.133, I63.139, I63.19, I63.2, I63.20, I63.21, I63.211, I63.212, I63.213, I63.219, I63.22, I63.23, I63.231, I63.232, I63.233, I63.239, I63.29, I63.3, I63.30, I63.31, I63.311, I63.312, I63.313, I63.319, I63.32, I63.321, I63.322, I63.323, I63.329, I63.33, I63.331, I63.332, I63.333, I63.339, I63.34, I63.341, I63.342, I63.343, I63.349, I63.39, I63.4, I63.40, I63.41, I63.411, I63.412, I63.413, I63.419, I63.42, I63.421, I63.422, I63.423, I63.429, I63.43, I63.431, I63.432, I63.433, I63.439, I63.44, I63.441, I63.442, I63.443, I63.449, I63.49, I63.5, I63.50, I63.51, I63.511, I63.512, I63.513, I63.519, I63.52, I63.521, I63.522, I63.523, I63.529, I63.53, I63.531, I63.532, I63.533, I63.539, I63.54, I63.541, I63.542, I63.543, I63.549, I63.59, I63.6, I63.8, I63.81, I63.89, I63.9 |
| Cerebrovascular disease | I65.0, I65.01, I65.02, I65.03, I65.09, I65.1, I65.2, I65.21, I65.22, I65.23, I65.29, I65.8, I66, I66.0, I66.01, I66.02, I66.03, I66.09, I66.1, I66.11, I66.12, I66.13, I66.19, I66.2, I66.21, I66.22, I66.23, I66.29, I66.3, I66.8, I66.9, I67, I67.0, I67.1, I67.2, I67.3, I67.4, I67.5, I67.6, I67.7, I67.8, I67.81, I67.82, I67.83, I67.84, I67.9 |
| Peripheral vascular disease | I73.9, I73.89, E08.5, E09.5, E10.5, E11.5, E13.5, I70.20, I70.21, I70.22, I70.26, I70.29, I70.23, I70.24, I70.25, I70.211, I70.212, I70.213, I70.218, I70.311, I70.312, I70.313, I70.318, I70.611 |
| Chronic lung disease | J40, J41, J41.0, J41.1, J41.8, J42, J43, J43.9, J44, J44.0, J44.1, J44.9, J45, J45.2, J45.20, J45.21, J45.22, J45.3, J45.30, J45.31, J45.32, J45.4, J45.40, J45.41, J45.42, J45.5, J45.50, J45.51, J45.52, J45.9, J45.90, J45.901, J45.902, J45.909, J45.99, J45.990, J45.991, J45.998, J47, J47.0, J47.1, J47.9 |
| Chronic kidney disease | N18, N18.1, N18.2, N18.3, N18.4, N18.5, N18.6, N18.9, N19 |
| Diabetes mellitus | E10, E11, E11.2, E11.21, E11.22, E11.29, E11.3, E11.31, E11.39, E11.32, E11.321, E11.329, E11.33, E11.331, E11.339, E11.34, E11.341, E11.349, E11.35, E11.351, E11.352, E11.353, E11.354, E11.355, E11.359, E11.36, E11.37, E11.39, E11.4, E11.40, E11.41, E11.42, E11.43, E11.44, E11.49, E11.5, E11.51, E11.52, E11.59, E11.6, E11.61, E11.610, E11.618, E11.62, E11.620, E11.621, E11.622, E11.628, E11.63, E11.630, E11.638, E11.64, E11.641, E11.649, E11.65, E11.69, E11.8, E11.9, E13 |
| Chronic liver disease / Cirrhosis | K70, K70.0, K70.1, K70.10, K70.11, K70.2, K70.3, K70.30, K70.31, K70.4, K70.40, K70.41, K70.9, K72.1, K72.10, K72.11, K72.9, K72.90, K72.91, K73, K73.0, K73.1, K73.2, K73.8, K73.9, K74, K74.0, K74.1, K74.2, K74.3, K74.4, K74.5, K74.6, K74.60, K74.69, K74.5, K75.9, K76.1, K76.9 |
| Dementia | F01, F01.5, F01.50, F02, F02.8, F02.80, F02.81, F03, F03.9, F03.90, F03.91, F04 |
| Frailty | R54, R53.0, R53.1, R53.2, R53.8, R53.81, R53.82, R41.81, R53.83 |
| Inability to walk | R26, R26.0, R26.1, R26.2, R26.8, R26.9, R27, R27.0, R27.8, R27.9, R29.3, R29.4, R29.6 |
| Depression | F32, F32.0, F32.1, F32.2, F32.3, F32.4, F32.5, F32.9, F33, F33.0, F33.1, F33.2, F33.3, F33.4, F33.40, F33.41, F33.42, F33.8, F33.9 |
| Malnutrition | E40, E41, E42, E43, E44, E44.0, E44.1, E45, E46 |
| Obesity | E66, E66.0, E66.01, E66.09, E66.1, E66.2, E66.3, E66.8, E66.9 |
| Prosthetic aortic valve | Z95.2 and Z95.3 |

**Supplementary table 3. Different age groups in each ADI group.**

| **Variable** | **Group A**  **ADI = 1, 2**  **(n = 301, %)** | **Group B**  **ADI = 3, 4**  **(n = 439, %)** | **Group C**  **ADI = 5, 6**  **(n = 436, %)** | **Group D**  **ADI = 7, 8**  **(n = 331, %)** | **Group E**  **ADI = 9, 10**  **(n = 244, %)** | ***P* value** |
| --- | --- | --- | --- | --- | --- | --- |
| Age group 40-59 | 10(3.3) | 15(3.4) | 23(5.3) | 30(9.1) | 26(10.7) | **0.0009** |
| Age group 60-80 | 126(41.9) | 179(40.8) | 164(37.6) | 128(38.7) | 113(46.3) |  |
| Age group >80 | 165(54.8) | 245(55.8) | 249(57.1) | 173(52.3) | 105(43) |  |
